# Feasibility and Acceptability of a Mobile Mindfulness Meditation Intervention Among Women: Intervention Study

**DOI:** 10.2196/15943

**Published:** 2020-06-02

**Authors:** Ariane Lisann Rung, Evrim Oral, Lara Berghammer, Edward S Peters

**Affiliations:** 1 Epidemiology Program School of Public Health Louisiana State University Health Sciences Center New Orleans New Orleans, LA United States; 2 Biostatistics Program School of Public Health Louisiana State University Health Sciences Center New Orleans New Orleans, LA United States; 3 Wounded Warrior Project San Diego, CA United States

**Keywords:** mindfulness, mobile phone, depressive symptoms, women, Louisiana

## Abstract

**Background:**

Traditional mindfulness-based stress reduction programs are resource intensive for providers and time- and cost-intensive for participants, but the use of mobile technologies may be particularly convenient and cost-effective for populations that are busy, less affluent, or geographically distant from skilled providers. Women in southern Louisiana live in a vulnerable, disaster-prone region and are highly stressed, making a mobile program particularly suited to this population.

**Objective:**

This study aimed to (1) assess the feasibility and acceptability of a mobile mindfulness app in real-world conditions in a pilot study of a community sample of women residing in southern Louisiana, (2) describe predictors of app usage, and (3) assess the effect of the app on secondary health outcomes.

**Methods:**

Women were recruited from an oil spill study on health. A total of 236 women completed a baseline survey, were offered the mobile mindfulness program, and completed a follow-up survey. Subjects were asked to download and use the app for at least 30 days for 10 min. All study procedures were completed on the web. Primary outcomes were feasibility and acceptability of the app and characteristics of app utilization. Secondary outcomes included mindfulness, depression, perceived stress, sleep quality, physical activity, BMI, and healthy eating.

**Results:**

Overall, 74.2% (236/318) of subjects completed the follow-up survey, and 13.5% (43/318) used the app. The main barrier to app usage was lack of time, cited by 37% (16/43) of users and 48.7% (94/193) of nonusers of the app. Women who chose to use the app were more highly educated (16/43, 63% had a college education vs 65/193, 33.7% of nonparticipants; *P*<.001), had higher incomes (23/43, 58% had incomes >US $50,000 per year vs 77/193, 43.0% of nonparticipants), and were employed (34/43, 79% vs 122/193, 63.2% of nonparticipants; *P*=.047). Those who engaged with the app did so at high levels, with 72% (31/43) of participants self-reporting the completion of some or all sessions and 74% (32/43) reporting high levels of satisfaction with the app. Participation with the app had a beneficial impact on depression (odds ratio [OR] 0.3, 95% CI 0.11-0.81), sleep quality (OR 0.1, 95% CI 0.02-0.96), sleep duration (OR 0.3, 95% CI 0.07-0.86), sleep latency (OR 0.3, 95% CI 0.11-0.81), and physical activity (2.8 95% CI 1.0-7.8), but mindfulness scores did not change from baseline to follow-up.

**Conclusions:**

The Headspace mobile mindfulness app was easy and cost-effective to implement and acceptable to those who participated, but few women elected to try it. The unique characteristics of this southern Louisiana population suggest that more intense promotion of the benefits of mindfulness training is needed, perhaps in conjunction with some therapist or researcher support. Several short-term benefits of the app were identified, particularly for depression and sleep.

## Introduction

### Background

Mindfulness refers to a state of consciousness that focuses on an individual’s attention and awareness in the present moment [1]. Mindfulness-based stress reduction (MBSR) is a standardized meditation program created from efforts to integrate Buddhist mindfulness meditation with contemporary clinical and psychological practice [2]. MBSR and other forms of mindfulness-based therapy have been observed in systematic reviews and meta-analyses to convey a variety of beneficial mental health outcomes, such as lowered anxiety, stress, and depression [3-5]. Other studies have also explored its effect on sleep quality [3,6,7], physical activity [8,9], smoking [9], and eating behaviors [9,10], although with mixed or inconclusive results.

Traditional MBSR programs can be resource intensive for providers and time- and cost-intensive for participants. Interventions typically consist of 8 weekly meetings led by a trained facilitator, with daily homework exercises and a weeklong retreat [2]. Individuals likely to benefit from this type of intervention may not have the time or resources to engage in such programs nor have easy access to experienced leaders. Thus, alternative low-intensity self-help methods are needed to expand the reach of traditional mindfulness-based approaches. Given that 77% of Americans now own a smartphone [11], the use of mobile technologies for this purpose may be particularly convenient and cost-effective for populations that are busy, less affluent, or geographically distant from skilled providers. Low-intensity [12] and web-based interventions [13] have begun to show promise in improving mindfulness, depression, and stress outcomes.

Southern Louisiana is a vulnerable, disaster-prone region with a highly stressed population. Recent disasters, such as the 2005 hurricanes and the 2010 Deepwater Horizon oil spill, have resulted in significant cumulative mental health impacts related to depression, anxiety, psychological distress, and posttraumatic stress disorder [14-17]. Mental health services were decimated following the 2005 hurricanes [16,18], resulting in residents receiving less mental health treatment than they required. Women, in particular, represent an influential yet vulnerable and understudied population. They are often central to decision-making processes within households, particularly with respect to decisions regarding health, support, diet, and caregiving. Therefore, there is a need for low-cost, easy-to-implement mental health and healthy lifestyle interventions that can be disseminated over large population areas. As MBSR has shown promising results, particularly in the area of stress reduction [5], a low-intensity mindfulness-based intervention may be a useful tool in the disaster recovery toolbox.

### Objectives

This study was designed to assess the use of a mobile mindfulness program under real-world conditions within a community sample of women residing in southern Louisiana. The objectives of this study were to (1) assess the feasibility and acceptability of a mobile mindfulness meditation program, (2) describe the predictors of program usage among study participants, and (3) assess the effect of the program on secondary health outcomes.

## Methods

### Participants

**Figure 1:**
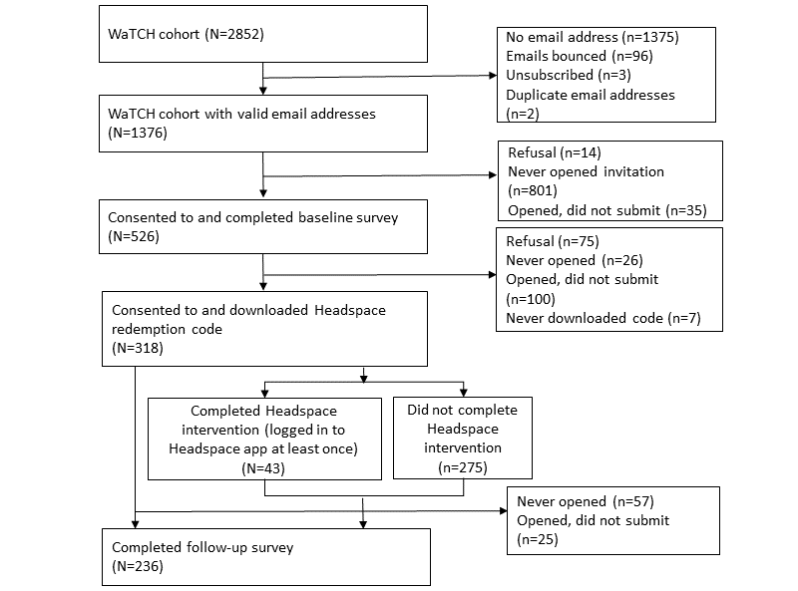
Study flowchart. WaTCH: Women and Their Children's Health.

Participants of the Women and Their Children’s Health (WaTCH) study, designed to investigate the physical and mental health effects of the Deepwater Horizon oil spill in Louisiana, were invited to enroll in the study. WaTCH participants (n=2852) were followed over 2 waves of data collection (2012-2016). Eligible women were aged 18 to 80 years at the time of initial data collection from 2012 to 2014 and resided in 1 of 7 parishes in southern Louisiana on April 20, 2010 [19]. A total of 1376 adult WaTCH participants who provided valid email addresses were invited to participate in a prospective pilot study on MBSR and stress among women during the summer of 2017. Of 1376 participants, 526 consented to and completed the baseline survey, of whom 318 consented to participate in the MBSR mobile mindfulness app component of the study, and 236 completed the follow-up survey and comprised the final sample. Figure 1 shows the study flow chart. The study was approved by the Louisiana State University Health Sciences Center (LSUHSC) New Orleans institutional review board.

### Procedure

#### Recruitment

Adult WaTCH participants were recruited through email invitation, consented through the web, and administered a web-based baseline survey via Research Electronic Data Capture hosted at the Epidemiology Data Center at the LSUHSC School of Public Health [20]. On completion of the baseline survey, subjects were asked to use a mobile mindfulness app (Headspace, described under *The Headspace Program*) and asked to indicate their consent by signing a data use privacy acknowledgment form. They were then given a redemption code for free access to the app.

During the study period, subjects had no interaction with the study team. Headspace has its own built-in reminders that can be programmed according to user specifications. After 45 days, the subjects were prompted to complete the follow-up survey. Subjects completed the follow-up survey an average of 80.7 (SD 49.2) days after the baseline survey.

#### Email Reminders

At each subsequent step of the study (ie, baseline survey consent, baseline survey, program consent, Headspace acknowledgment, and follow-up survey), those who had not completed the task were sent periodic automated reminders every 4 days until task completion, up to 3 times. For the follow-up survey, automated reminders were sent up to 5 times. A one-time email reminder was also sent automatically 7 days after the Headspace acknowledgment was signed to remind the subjects to complete the program. The study staff had no information on whether subjects had downloaded or used the app until after study completion.

#### Incentives

Subjects were given US $10 on completion of the baseline survey and US $10 on completion of the follow-up survey. Those who completed the Headspace acknowledgment form were also given free access to Headspace for 1 year (value approximately US $96).

### The Headspace Program

The mobile MBSR training program consisted of using a smartphone or web-based app called Headspace. Headspace was selected because it had the highest average rating in a review of 23 apps that provided mindfulness training and education [21]. Subjects were asked to download the app and use it for at least 30 days, 10 min at a time. After 45 days, they were reminded to complete a web-based follow-up survey. The app contained a *Foundations* series consisting of 3 groups of 10 sessions at the *Basics* level as well as a variety of other themed packs. Most sessions were designed to be used for 10 min per day. Participants had the ability to explore the app and complete any of the other sessions they desired for a full year. Although the study staff had no formal contact with participants during the study period, they were available to provide technical support on request (via an email or a toll-free number). On completion of the data collection period, Headspace developers provided the researchers with participant data on number of sessions, date/time of meditation sessions attempted, and platform uses.

### Measures

The baseline and follow-up surveys consisted of questions about participant demographics, physical and mental health, and social and environmental characteristics. The follow-up survey also contained questions related to acceptability of the Headspace app program.

#### Primary Outcomes

The primary outcomes of the study were feasibility, acceptability, and characteristics of app utilization. *Feasibility* was defined as (1) enrollment (eligible subjects who consented to the study and completed the Headspace acknowledgment form), (2) program participation (enrolled subjects who logged into Headspace at least once during the study period), and (3) retention (enrolled subjects who completed the follow-up survey). *Acceptability* was measured through a series of closed-ended questions about how well participants liked the app. Example questions included “How would you rate the Headspace app on a scale of 1 to 5?” “Would you recommend Headspace to others?” and “How much of the Headspace program did you complete?” All subjects were also asked, “What do you think were the biggest barriers to completing the Headspace program?” Example responses that subjects were allowed to choose from included “not enough time,” “not interested in mindfulness meditation,” “didn’t see how mindfulness meditation would benefit me,” “no privacy or quiet space to do the meditation,” “did not like the guy’s voice,” “didn’t have access to a smartphone or computer every day,” and “technical problems.” Subjects were allowed to select as many responses as applied, including an option to specify something different. Data on *characteristics of* *Headspace app usage* included the total number of log-ins to Headspace, average log-ins per program completer, platform used (iOS, Android, or web-based), day of week of use (weekday vs weekend), and time of day of use (in 4-hour blocks).

#### Secondary Outcomes

The secondary outcomes of the study included trait mindfulness, depressive symptoms, perceived stress, sleep quality, physical activity, body mass index (BMI), and healthy eating.

##### Mindfulness

Mindfulness was measured using the 15-item Mindful Attention Awareness Scale (MAAS), trait version, and ranged from 1=almost always to 6=almost never [1]. MAAS scores were averaged and dichotomized at the median.

##### Depressive Symptoms

Depressive symptoms were measured for the past week with the Center for Epidemiologic Studies Depression Scale-10 (CESD-10) [22]. Respondents rated the frequency of symptoms that occurred during the past week on a 4-point scale, ranging from 0=none of the time to 3=most of the time. Item scores were summed after reverse coding the positive mood items (range 0-30). They were then dichotomized such that total scores of 10 or greater were indicative of depressive symptoms [22].

##### Perceived Stress

Perceived stress was measured using the Perceived Stress Scale, 4-item version (PSS-4) [23], with responses ranging from 0=never to 4=very often. Items were summed after reverse scoring the positively worded items (range 0-16), with higher scores indicating greater levels of stress. PSS-4 scores for this study were dichotomized at the median.

##### Sleep Quality

Sleep quality was measured using the Pittsburgh Sleep Quality Index (PSQI), a 19-item self-rated questionnaire that assesses sleep quality and disturbances over a 1-month time interval [24]. A total of 7 component scores were created: subjective sleep quality, sleep latency (length of time it takes to fall asleep), sleep duration, habitual sleep efficiency (the percentage of time in bed that one is asleep), sleep disturbances, use of sleeping medication, and daytime dysfunction. The sum of the component scores yielded one global score (range 1-19), where higher scores indicate more sleep problems. A global score ≤5 is indicative of good sleep quality. Responses to the individual components of the index were grouped into 4 categories, ranging from 0 (better) to 3 (worse), and then dichotomized into 2 groups.

##### Physical Activity

Physical activity was measured using the Total Physical Activity Screener from the Stanford Brief Activity Survey [25]. In total, 2 questions asked about on-the-job activity and leisure-time activity. Responses were categorized into 5 levels of physical activity, from inactive to very hard intensity, and further dichotomized into inactive/light-intensity and moderate/hard/very hard–intensity activity.

##### Body Mass Index

BMI was calculated from self-reported height and weight, using the formula of weight in kilograms divided by height in meters squared, and then grouped into 2 groups: normal/underweight versus overweight/obese.

##### Healthy Eating

Healthy eating was assessed using items from the Dietary Screener of the 2009 California Health Interview Survey [26], which gathers information about the intake of fruits and vegetables and teaspoons of added sugar. In total, 2 summary measures were calculated: daily cup equivalents of fruits and vegetables and daily teaspoons of added sugar. Each measure was dichotomized at the median.

#### Other Covariates

Age at the time of the interview, race/ethnicity, household income, marital status, employment status, and number of minor children living in the household were also measured.

### Analysis

Descriptive statistics were calculated for all measures. Feasibility was assessed by calculating the enrollment percentage, program participation, and retention. Characteristics of those who consented to the program (N=318) were compared with those who completed both surveys (N=236). Baseline characteristics and per-protocol results were assessed. Comparisons of secondary outcomes between program participants and nonparticipants were performed using Pearson chi-square or Fisher exact tests for categorical variables. Multivariable logistic regression modeling was used to assess the associations between the secondary outcomes at follow-up and participation in the Headspace program. When needed, Firth penalized logistic regression models were used to overcome separation issues [27]. As the corresponding outcome at baseline and the total number of days each subject used the Headspace app were identified as potential confounders for the majority of secondary outcomes (ie, the crude and adjusted measures of association differed appreciably [28]), all regression models were adjusted for both variables. All statistical tests were carried out using SAS 9.3 (SAS Institute Inc) at a type 1 error level of 0.05.

## Results

### Feasibility

Of 526 women who were eligible to participate in the program because they had completed the baseline survey, 318 consented and completed the Headspace acknowledgment form, resulting in an enrollment of 60.5% of the eligible sample. Of the 318 women who enrolled, 43 (13.5%) actually participated in the program. Of those who enrolled, 236 women completed the follow-up survey, yielding a retention proportion of 74.2%.

### Baseline Characteristics of the Sample

Table 1 shows the demographic characteristics of the women in the sample. Women who consented to the study were similar to women who consented and completed follow-up measures. Most women who completed follow-up measures were white (140/236, 59.3%), had less than a college education (144/236, 61.0%), had a household income of less than US $50,000 per year (119/236, 50.4%), and were currently working full time or part time (156/236, 66%). Women who participated in the program were more likely to have a college education (27/43, 63% vs 65/193, 33.6%; *P*<.001) and be currently working outside the home (34/43, 79% vs 122/193, 63.2%; *P*=.047) than women who did not participate.

**Table 1:** Baseline demographic characteristics of the sample by program participation, Louisiana, 2017 to 2018.

Characteristics		Total sample^a^ (N=236)	Program participants^a^ (N=43)	Nonparticipants^a^ (N=193)	*P* value	Consent only^a^ (N=318)
**Race/ethnicity^b^, n (%)**					.19	N/A^c^
	Non-Hispanic white	140 (59.3)	29 (67.4)	111 (57.5)	N/A	165 (51.8)
	Non-Hispanic black or other/multi/Hispanic	93 (39.4)	13 (30.2)	80 (41.4)	N/A	137 (43.0)
**Education, n (%)**					<.001	N/A
	High school graduate or less	144 (61.0)	16 (37.2)	128 (66.3)	N/A	195 (61.3)
	College or more	92 (38.9)	27 (62.7)	65 (33.6)	N/A	122 (38.3)
**Current household income^b^, US $, n (%)**					.10	N/A
	≤50,000 per year	119 (50.4)	17 (39.5)	102 (52.8)	N/A	165 (51.8)
	>50,000 per year	100 (42.3)	23 (53.4)	77 (39.8)	N/A	134 (42.1)
**Marital status, n (%)**					.32	N/A
	Married or living with a partner	149 (63.1)	30 (69.7)	119 (61.6)	N/A	203 (63.8)
	Widowed, divorced, separated, or never married	87 (36.8)	13 (30.2)	74 (38.3)	N/A	115 (36.1)
**Employment status, n (%)**					.047	N/A
	Currently working full time or part time	156 (66.1)	34 (79.0)	122 (63.2)	N/A	211 (66.3)
	Not currently working full time or part time	80 (33.8)	9 (20.9)	71 (36.7)	N/A	107 (33.6)
Age^b^ (years), mean (SD)		46.1 (10.0)	46.6 (9.8)	46.0 (10.1)	.82	46.8 (10.3)
Number of children aged <18 years living in a household, mean (SD)		1.1 (1.2)	0.8 (0.9)	1.2 (1.3)	.18	1.1 (1.2)

^a^Total sample (N=236) includes those who completed both the baseline and follow-up surveys. Program participants (N=43) include program completers, those who logged into the Headspace app at least once and completed both surveys. Nonparticipants (N=193) include program noncompleters. Consent only (N=318) includes those who completed the baseline survey and consented to the program but did not complete the follow-up survey.

^b^Race/ethnicity missing (n=3); income missing (n=17); and age missing (n=1).

^c^N/A: not applicable.

### Acceptability of Headspace App

The acceptability of the program was assessed among the 43 women who used the app and subsequently completed the follow-up survey (Table 2). Most log-ins (1191/1530, 77.8%) took place on an iOS device and on a weekday (1147/1530, 75.0%). Most sessions (375/1530, 24.5%) were conducted in the afternoons between noon and 4 PM, and another 20.3% (310/1530) of sessions were conducted in the early evenings between 4 PM and 8 PM. Women logged into the app, on average, 36 times (SD 80) for an average of 24 days (SD 36). This includes one enthusiastic participant who logged in a total of 503 times and another who logged in over 156 days.

Participants who used the Headspace app were also asked about their experiences with it. Almost three-fourth of participants reported that they were pleased with it or loved it, and more than 85% said they would recommend the app to others. More than two-third of participants reported that they had completed at least some or all of the program. More than two-third of participants said they liked the relaxation aspect of Headspace the best, followed by the voice of the meditation leader, and the duration of the session.

Participants (N=43) and nonparticipants (N=193) were also asked to report what they did not like about the Headspace app (Table 3). The biggest barriers cited among those who managed to participate were lack of time (16/43, 37%), lack of privacy (8/43, 19%), and lack of a quiet space to meditate (8/43, 19%). Among those who did not participate, the biggest reported barriers were time (94/193, 48.7%), lack of a quiet space (26/193, 13.5%), and lack of privacy (21/193, 10.9%).

**Table 2:** Characteristics of Headspace usage among participants.

Characteristics			Values
**Access characteristics per log-in (N=1530), n (%)**			
	**Platform used**		
		iOS	1191 (77.8)
		Android	116 (7.6)
		Desktop	223 (14.6)
	**Day of week log-in session occurred**		
		Weekday	1147 (75.0)
		Weekend	383 (25.0)
	**Time of day log-in session began**		
		Midnight to 4 AM	301 (19.7)
		4 AM to 8 AM	243 (15.9)
		8 AM to noon	158 (10.3)
		Noon to 4 PM	375 (24.5)
		4 PM to 8 PM	310 (20.3)
		8 PM to midnight	143 (9.3)
**Access per participant (N=43), mean (SD; range)**			
		Log-ins to Headspace	35.6 (80.3; 1-503)
		Number of days used Headspace	24.0 (36.1; 1-156)
**Acceptability per participant (N=43), n (%)**			
	**Overall rating of Headspace app**		
		Hated it	1 (2.3)
		Not crazy about it	6 (14.0)
		Feel neutral about it	4 (9.3)
		Pleased with it	19 (44.2)
		Loved it	13 (30.2)
	**Would recommend Headspace app to others**		
		Yes	37 (86.1)
		No	6 (14.0)
	**How much of Headspace completed**		
		None	0 (0.0)
		A little	12 (27.9)
		Some	15 (34.9)
		All	16 (37.2)
	**What did you like best about the Headspace program?**		
		Relaxation	12 (36.4)
		Voice	5 (15.2)
		Good length of time	4 (12.1)
		Good concept	3 (9.1)
		Forced me to take personal time	3 (9.1)
		Easy access	2 (6.1)
		Completed	1 (3.0)
		Slept better	1 (3.0)
		Daily reminders	1 (3.0)
		Effective program	1 (3.0)

**Table 3:** Barriers to Headspace use.

Characteristics	Participants (N=43), n (%)	Nonparticipants (N=193), n (%)
Not enough time	16 (37.2)	94 (48.7)
No privacy to do the meditation	8 (18.6)	21 (10.9)
No quiet space to do the meditation	8 (18.6)	26 (13.5)
Did not like the guy's voice on the Headspace app	7 (16.3)	7 (3.6)
Not interested in mindfulness meditation	3 (7.0)	19 (9.8)
Didn’t see how mindfulness meditation would benefit me	3 (7.0)	16 (8.3)
Technical problems with installation or use	0 (0.0)	12 (6.2)
Didn’t have access to a smartphone or computer every day	1 (2.3)	17 (8.8)

### Effect of the Headspace Program on Secondary Outcomes

A description of the secondary outcomes at baseline is shown in Multimedia Appendix 1. Data are presented for the total sample of those who completed both surveys (N=236), for the program participants (N=43) and nonparticipants (N=193), and for those who consented but did not complete the program or the follow-up survey. At baseline, almost half of the study sample (111/227, 48.9%) reported high levels of depressive symptoms, 59.8% (141/236) reported high levels of perceived stress, 64.7% (141/218) reported high levels of sleep problems, 81.7% (183/224) reported BMI in the overweight or obese category, and 50.3% (94/187) reported high levels of sugar intake. In terms of healthy behaviors, 50.4% (115/227) of the study sample reported high levels of mindfulness 36.8% (86/234) reported high levels of physical activity, and 50.0% (114/228) reported high levels of daily fruit and vegetable intake. Program participants and nonparticipants tended to have similar scores on all outcomes at baseline, as did the group that only consented.

The results from logistic regression models predicting the effect of Headspace program participation on secondary outcomes at follow-up are shown in Table 4. All models were adjusted for condition at baseline and the total number of days the app was used. Depressive symptoms, sleep quality, sleep duration, sleep latency, physical activity, and fruit and vegetable intake all improved after participation in the Headspace program. Those who participated in the Headspace program were 0.3 (95% CI 0.11-0.81) times likely to be depressed at follow-up, 0.1 (95% CI 0.02-0.96) times likely to have poor sleep quality, 0.3 (95% CI 0.07-0.86) times likely to have poor sleep duration, 0.3 (95% CI 0.12-0.99) times likely to have poor sleep latency, 2.8 (95% CI 1.0-7.8) times likely to participate in moderate to very hard physical activity, and 0.94 (95% CI 0.99-5.78) times likely to have increased fruit and vegetable intake. No changes at follow-up were observed for mindfulness or other health indicators.

**Table 4:** Individual logistic regression models predicting the effect of the program on outcome at follow-up, adjusted for number of days app was used, and outcome at baseline.

Characteristic		Participants, N	Odds ratio (95% Wald confidence limits)	*P* value
Greater mindfulness (MAAS^a^ ≥4.13)		219	1.69 (0.53-5.38)	.37
More depressive symptoms (CESD-10^b^ ≥10)		224	0.29 (0.11-0.81)	*.*02
Greater perceived stress (PSS^c^ ≥6)		235	0.76 (0.31-1.85)	.55
**PSQI^d^**				
	Poor habitual sleep efficiency	196	1.56 (0.52-4.64)	.43
	Poor sleep quality	196	0.14 (0.02-0.96)	.045
	Need for medications to sleep	196	0.47 (0.13-1.70)	.25
	Poor sleep duration	196	0.25 (0.07-0.86)	.03
	Sleep disturbance	196	1.04 (0.41-2.68)	.93
	Poor sleep latency	196	0.34 (0.12-0.99)	.048
	Day dysfunction due to sleepiness	196	0.44 (0.14-1.41)	.17
	Total PSQI score >5	196	2.35 (0.63-8.77)	.20
Physical activity (moderate/hard/very hard intensity)		221	2.79 (1.00-7.78)	.05
BMI^e^ (overweight/obese)^f^		216	0.52 (0.06-4.67)	.56
**Healthy eating measures**				
	Fruit and vegetable intake ≥0.91 daily cup equivalents	201	0.94 (0.99-5.78)	.05
	Sugar intake ≥6.25 teaspoons	160	1.00 (0.35-2.86)	.99

^a^MAAS: Mindful Attention Awareness Scale.

^b^CESD-10: Center for Epidemiologic Studies Depression Scale-10.

^c^PSS: Perceived Stress Scale.

^d^PSQI: Pittsburgh Sleep Quality Index.

^e^BMI: body mass index.

^f^Firth penalized logistic regression model used to overcome separation issues.

## Discussion

The objectives of this study were to (1) assess the feasibility and acceptability of a mobile mindfulness meditation program among women residing in southern Louisiana, (2) describe the predictors of program usage among study participants, and (3) assess the effect of the program on secondary health outcomes.

### Feasibility, Acceptability, and Predictors of App Usage

#### Retention

In this study, 74% (32/43) of the participants completed the follow-up survey. A systematic review and meta-analysis of mindfulness self-help interventions, including delivery through the web, reported that an average of 73% of randomized participants completed posttreatment measures, which is comparable with attrition in studies of other self-help and minimal contact therapies [12]. This study demonstrated similar findings, suggesting that it is, although not a randomized trial, within the general norms for study completion.

#### Program Participation

However, only 13.5% (43/318) of participants in this study actually participated in the program, which was broadly defined as logging in to the Headspace app at least once. This is substantially lower than the participation rates demonstrated in similar studies. A review of self-help mindfulness intervention randomized controlled trials (RCTs) found that 48% of participants met the study-defined intervention engagement criteria [12]. Similarly, over a dozen studies to date have been published evaluating the feasibility or effectiveness of Headspace, and many of these also have stronger participation rates than this study. This calls into question whether Headspace is really a feasible intervention for this particular population of women. The majority of Headspace studies employed samples of university students or residents [29-35], samples of employees [36,37], or clinical samples [38,39]. Only 3 studies of Headspace used community samples, and these participants were predominantly self-selecting, white, well-educated [40,41], and living in Australia [40] or the United Kingdom [42]. This contrasts with the sample of this study, which was recruited from a cohort of women representative of the area, of whom 35% were African Americans, only 39% had a college education or higher, and which took place in southern coastal Louisiana, an area that has been subject to quite a few natural and man-made disasters in recent years. Although our sample may have been more highly stressed than national norms [43], they were not recruited into the study to address any particular clinical condition nor were they offered any suggestions that the program would help them with their own issues, as might happen with a sample of participants recruited from a clinic. Thus, their perceived need to engage with the app may have been less. In addition, evidence suggests that clinical or therapist support is beneficial for promoting adherence in behavioral web-based interventions [12,44]. Prior studies of Headspace incorporated some in-person contact between participants and study personnel, either in the form of an initial briefing session [29,30,37] or by completing some sessions in a group setting [32,34]. However, most of these studies were interested in taking advantage of the completely self-directed nature of Headspace and learning how effective the app could be when administered entirely on the web with little engagement from study personnel. It is possible that this particular population, consisting of a less educated and significantly minority population, will require more intense personal encouragement to persuade them to use the app. Future interventions could consider including some type of nonautomated or in-person support. Another possibility for the low level of engagement may be because of study fatigue. Since 2012 and before this study, WaTCH participants had been asked to take part in two telephone surveys and a home visit, not to mention other research studies that were taking place in the area at the same time.

#### Barriers

The main barrier to using the Headspace app was lack of time, cited by both participants and nonparticipants alike, a finding that is echoed in the literature. A qualitative study of Headspace users found that the main concern of users was fitting the app into their busy lives [45], whereas a study of another wellness mobile app reported that being busy made it difficult to find suitable, peaceful moments to engage in the intervention exercises [46]. Mobile-based interventions, by virtue of their ability to be used anywhere at any time, are designed to address this concern, but competing demands on participants’ time make this a continuing challenge.

#### Predictors of App Usage and Acceptability

Differences between participants and nonparticipants may shed light on who is most likely to adopt the program and thus who the program is most likely to benefit. Those who chose to engage with the program tended to be more highly educated, have higher incomes, and be employed compared with those who did not, characteristics similar to those found in a national study of mindfulness practices [47]. It may be that women with these characteristics are more familiar with meditation practices in general and therefore more likely to use the Headspace app. Alternatively, it may be that the more educated and higher-income women are more likely to use mobile devices, as ownership of smartphones increases with education and income [11]. As the use of mobile devices is likely to keep increasing, additional promotion of the benefits of mindfulness practice may be warranted. Women who chose to use the app also tended to be less stressed. This suggests that those who perhaps need the program the most will require more persuasion to get them to use it.

Nevertheless, those who engaged with the app did so at high levels, self-reporting the completion of some if not all the sessions as well as high levels of satisfaction with the app. These findings highlight the importance of understanding the characteristics of those who choose to use the app [48], suggesting that it is an acceptable program for reaching large numbers of individuals.

### Secondary Outcomes

This study also explored the impact of mobile mindfulness-based program participation on a number of secondary health and behavioral outcomes. Participation had a beneficial impact on depressive symptoms, sleep indicators, and physical activity, but mindfulness scores did not change from baseline to follow-up.

#### Depression

Almost half of the women in our community sample reported symptoms of depression. A study among older adults, using the same scale and cutoff values, demonstrated the prevalence of depressive symptoms between 12.3% and 16.3% [22], providing more evidence that our own population may be highly stressed. Subjects who used the Headspace app were less likely to experience depressive symptoms at follow-up than women who did not use it. Most of the comprehensive reviews of depression and MBSR conducted in clinical samples using noninternet-based interventions [3,4,49,50] have demonstrated improvements in depression following MBSR, and studies in both clinical and community samples using internet- or mobile-based delivery have also noted decreases in depressive symptoms [37,51-53]. That we were able to detect similar improvements in our community sample, although uptake of the app was low, suggests that this may be a viable program to improve depressive symptoms in this population.

#### Sleep

A large proportion of our study participants reported sleep problems, particularly in the areas of sleep latency and sleep disturbance. Population-based studies of insomnia suggest that approximately 30% of the general population has complaints of sleep disruption, with female gender and older age being the predominant demographic risk factors [54]. Depression is one of the most common comorbid psychiatric disorders in insomniacs [55], whereas other consequences of sleep difficulties include decreased quality of life, increased accidents, and reduced work productivity [55]. Given the high prevalence of depression and sleep problems in our sample, the search for effective interventions could be critical. In our study, 3 components of the PSQI showed improvement after participants used the Headspace app: self-reported sleep quality, sleep duration, and sleep latency. The evidence for a beneficial effect of mindfulness programs on sleep in the literature is inconclusive. One review of studies found that MBSR significantly improved measures of sleep quality or duration [6], but 2 later reviews either found insufficient [3] or mixed evidence [7]. However, none of these reviews looked specifically at internet-based programs. Our results suggest that using Headspace may be beneficial for sleep, but more research in larger populations is warranted.

#### Physical Activity

Physical Activity Guidelines for Americans recommends at least 150 min per week of moderate-intensity physical activity [56], yet fewer than half of our sample reported being in at least the moderate-intensity category over the past year. Those who used the app were borderline more likely to see improvements in their physical activity levels after the program than those who did not. This is consistent with a systematic review of RCTs finding that mindfulness training had a positive effect on physical activity levels [57] and a review of cross-sectional studies indicating positive relationships between dispositional mindfulness and physical activity [58]. Moreover, results from the 2012 National Health Interview Survey showed that US adults who practiced mindfulness meditation in the past year were less likely to be inactive and more likely to meet physical activity recommendations [59]. The findings from this study indicate that using the Headspace app may be beneficial for physical activity, but more research in a larger population is warranted.

#### Mindfulness

MBSR is thought to improve certain health outcomes through its improvement of individuals’ levels of dispositional mindfulness [60-62]. A positive relationship between the program and levels of mindfulness would be a first step in testing whether this mechanism improves health. In this study, however, despite experiencing improvement in some secondary outcomes, participants did not significantly improve their mindfulness scores. Evidence in the scientific literature that self-reported mindfulness is a primary mechanism of change is mixed [63,64], and some have questioned the validity of these measures, including the MAAS used in this study [65]. Among the arguments posed are the lack of a clear gold standard with which to define a mindful person, the lack of consensus about what mindfulness is, and a debate about whether individuals can accurately self-report their own levels of mindfulness [65]. The MAAS instrument used to assess mindfulness in this study is a well-regarded and validated instrument [1], yet our results call into question whether self-reported mindfulness is indeed the actual mechanism through which the program improves health outcomes. Other potential reasons for the lack of significant associations between the program and mindfulness may be related to differences in duration and dosage of the intervention, characteristics of our sample population, and differences in levels of motivation to participate.

### Conclusions

This study was designed to assess the use of a mobile mindfulness intervention in real-world conditions in a community sample of women residing in southern Louisiana. The all-electronic program was easy and cost-effective to implement and acceptable to those who participated, but few women elected to try it. The unique characteristics of this population (more prone to environmental disasters, more minorities, less educated, and lower income) suggest that more intense promotion of the benefits of mindfulness training is needed, perhaps in conjunction with some therapist or researcher support. Several short-term benefits of the program were identified, particularly for depression and sleep.

#### Limitations

Some limitations of the study were identified: (1) No control group was used, making it difficult to determine whether the observed effects were because of the use of the app or other characteristics of the sample that led them to choose to engage with the app. It is possible that participants who already believed in the benefits of meditation were more likely to choose to use the app and to report that they benefited from it than those who did not. (2) The length of time spent in mindfulness sessions was not clearly defined in this study. Participants were asked to spend at least 10 min per day using the app, but the researchers did not have access to specific data reporting the exact length of time each participant actually spent with the app. More information regarding *dosage* would help inform how much mindfulness exposure is needed to achieve benefits. (3) Participants were asked to use the app only for a period of 30 days, although they had access to it for much longer. Although we adjusted for the number of days participants used the app, meditation is a skill that many spend a lifetime learning and that traditional MBSR programs teach for longer periods. The length of exposure in this study may not have been sufficient to detect changes in secondary outcomes. (4) The sample was exclusively female, limiting the generalizability to men. (5) The sample differed from the original WaTCH cohort in that they were slightly younger and were more likely to be college educated, have a higher income, and be working full time than the original cohort. (6) The incentive structure of the study resulted in subjects being paid for completing both surveys, but not being paid for participating in the actual mindfulness program itself. This may have led to a potential bias in the composition of the participant and nonparticipant groups, as participants tended to be better educated and employed than nonparticipants.

#### Strengths

The strengths of this study include (1) the setting of the program in southern Louisiana, within a rural population residing in a low-density area where access to group-based in-person mindfulness approaches is sparse and likely not sustainable; (2) the population was a sample of women residing in the community, without specific clinical manifestations, and thus addresses the need to translate prior research into effective and sustainable community mindfulness intervention programs [63]; (3) the sample contained individuals from a variety of racial, educational, and socioeconomic backgrounds that are traditionally underrepresented among mindfulness practitioners. As the study was not an RCT, it was able to highlight some important differences between individuals who chose to engage with the app and those who did not, namely, in terms of income, education, and stress levels; (4) the study used mobile technology to implement a self-help program approach to mindfulness, addressing issues such as lack of access to facilities and instructors, resources, and time to meditate.

This study demonstrated the feasibility and acceptability of implementing a mobile mindfulness meditation program in a population of women in southern Louisiana. Such apps represent a convenient and cost-effective technology that can easily be scaled up to address barriers to the implementation of traditional MBSR programs but may require supplemental support to promote their use.

## Appendix

Multimedia Appendix 1 Baseline measurement of secondary outcomes by program participation, Louisiana, 2017 to 2018.

## Abbreviations

CESD-10: Center for Epidemiologic Studies Depression Scale-10
LSUHSC: Louisiana State University Health Sciences Center
MAAS: Mindful Attention Awareness Scale
MBSR: mindfulness-based stress reduction
OR: odds ratio
PSQI: Pittsburgh Sleep Quality Index
PSS-4: Perceived Stress Scale, 4-item version
RCT: randomized controlled trial
WaTCH: Women and Their Children’s Health
