# Supplementary material for: Feasibility and Acceptability of a Mobile Mindfulness Meditation Intervention Among Women: Intervention Study
Source: JMIR Mhealth Uhealth. 2020 Jun 2;8(6):e15943. doi: 10.2196/15943 (PMC7298633; doi:10.2196/15943)
Supplement: Multimedia Appendix 1 [file mhealth_v8i6e15943_app1.docx]

**Multimedia Appendix 1.** Baseline measurement of secondary outcomes by program participation, Louisiana, 2017 to 2018.^b^

| Characteristic | | | Total sample^a^ (N=236) , n (%) | Program participants^a^ (N=43), n (%) | Nonparticipants^a^ (N=193), n (%) | *P* value | Consent only^a^ (N=318), n (%) |
| --- | --- | --- | --- | --- | --- | --- | --- |
| **Mindfulness** | | | | | | .95 |  |
|  | | Higher levels (MAAS^c^ ≥4.13) | 115 (50.4) | 21 (50.0) | 94 (50.5) |  | 135 (44.3) |
|  | | Lower levels (MAAS <4.13) | 113 (49.6) | 21 (50.0) | 92 (49.5) |  | 170 (55.7) |
| **Depressive symptoms** | | | | | | .99 |  |
|  | | No depressive symptoms (CESD-10^d^ <10) | 116 (51.1) | 22 (51.2) | 94 (51.1) |  | 156 (51.2) |
|  | | More depressive symptoms (CESD-10 ≥10) | 111 (48.9) | 21 (48.8) | 90 (48.9) |  | 149 (48.9) |
| **Perceived stress** | | | | | | .20 |  |
|  | | Lower perceived stress (PSS^e^ <6) | 95 (40.3) | 21 (48.8) | 74 (38.3) |  | 125 (39.4) |
|  | | Greater perceived stress (PSS ≥6) | 141 (59.8) | 22 (51.2) | 119 (61.7) |  | 192 (60.6) |
| **PSQI^f^** | | | | | | | |
|  | **Habitual sleep efficiency** | | | | | .50 |  |
|  |  | Good habitual sleep efficiency | 172 (78.9) | 30 (75.0) | 142 (79.8) |  | 222 (76.6) |
|  |  | Poor habitual sleep efficiency | 46 (21.1) | 10 (25.0) | 36 (20.2) |  | 68 (23.5) |
|  | **Overall sleep quality** | | | | | .25 |  |
|  |  | Good sleep quality | 141 (64.7) | 29 (72.5) | 112 (62.9) |  | 185 (63.8) |
|  |  | Poor sleep quality | 77 (35.3) | 11 (27.5) | 66 (37.1) |  | 105 (36.2) |
|  | **Need medications to sleep** | | | | | .83 |  |
|  |  | Lesser need for medications | 161 (73.9) | 29 (72.5) | 132 (74.2) |  | 211 (72.8) |
|  |  | Greater need for medications | 57 (26.2) | 11 (27.5) | 46 (25.8) |  | 79 (27.2) |
|  | **Sleep duration** | | | | | .58 |  |
|  |  | Good sleep duration | 155 (71.1) | 27 (67.5) | 128 (71.9) |  | 200 (69.0) |
|  |  | Poor sleep duration | 63 (28.9) | 13 (32.5) | 50 (28.1) |  | 90 (31.0) |
|  | **Sleep disturbance** | | | | | .73 |  |
|  |  | Less sleep disturbance | 87 (39.9) | 15 (37.5) | 72 (41.5) |  | 112 (38.6) |
|  |  | More sleep disturbance | 131 (60.1) | 25 (62.5) | 106 (59.6) |  | 178 (61.4) |
|  | **Sleep latency** | | | | | .73 |  |
|  |  | Better sleep latency | 109 (50.0) | 21 (52.5) | 88 (49.4) |  | 144 (49.7) |
|  |  | Poor sleep latency | 109 (50.0) | 19 (47.5) | 90 (50.6) |  | 146 (50.3) |
|  | **Day dysfunction due to sleepiness** | | | | | .09 |  |
|  |  | Less day dysfunction | 145 (66.5) | 22 (55.0) | 23 (69.1) |  | 193 (66.6) |
|  |  | More day dysfunction | 73(33.5) | 18 (45.0) | 55 (30.9) |  | 97 (33.5) |
|  | **Total sleep index score** | | | | | .75 |  |
|  |  | Better (PSQI score ≤5) | 77(35.3) | 15 (37.5) | 62 (34.8) |  | 92 (31.7) |
|  |  | Worse (PSQI score >5) | 141 (64.7) | 25 (62.5) | 116 (65.2) |  | 198 (68.3) |
|  | **Physical activity** | | | | | .84 |  |
|  |  | Moderate/hard/very hard intensity | 86 (36.8) | 16 (38.1) | 70 (36.5) |  | 111 (35.2) |
|  |  | Inactive/light intensity | 148 (63.3) | 26 (61.9) | 122 (63.5) |  | 204 (64.8) |
|  | **BMI (overweight/obese)** | | | | | .50 |  |
|  |  | Normal/underweight | 41 (18.3) | 9 (22.0) | 32 (17.5) |  | 53 (17.7) |
|  |  | Overweight/obese | 183 (81.7) | 32 (78.1) | 151 (82.5) |  | 247 (82.3) |
| **Healthy eating measures** | | | | | | |  |
|  | **Fruit and vegetable intake (daily cup equivalents)** | | | | | .50 |  |
|  |  | ≥0.91 daily cup equivalents | 110 (50.2) | 22 (55.0) | 88 (49.2) |  | 142 (49.3) |
|  |  | <0.91 | 109 (49.8) | 18 (45.0) | 91 (50.8) |  | 146 (50.7) |
|  | **Sugar intake** | | | | | .28 |  |
|  |  | <6.25 teaspoons | 93 (49.7) | 15 (41.7) | 78 (51.7) |  | 126 (51.2) |
|  |  | ≥6.25 teaspoons | 94 (50.3) | 21 (58.3) | 73 (48.3) |  | 120 (48.8) |

^a^Total sample (N=236) includes those who completed both the baseline and follow-up surveys. Program participants (N=43) include program completers, those who logged into the Headspace app at least once and completed both surveys. Nonparticipants (N=193) include program noncompleters. Consent only (N=318) includes those who completed the baseline survey and consented to the program but did not complete the follow-up survey.

^b^Missing values: depressive symptoms (n=9); mindfulness (n=8); physical activity (n=2); PSQI variables (n=18); BMI (n=12); days drank in past year (n=24); usual number of drinks per day (n=5); binge drink (n=2); CAGE (n=2); fruit and vegetable intake (n=16); fruit and vegetable intake excluding fried potatoes (n=7); and sugar intake (n=49).

^c^MAAS: Mindful Attention Awareness Scale.

^d^CESD-10: Center for Epidemiologic Studies Depression Scale-10.

^e^PSS: Perceived Stress Scale.

**^f^**PSQI: Pittsburgh Sleep Quality Index.
